# Discovery and Targeted LC-MS/MS of Purified Polerovirus Reveals Differences in the Virus-Host Interactome Associated with Altered Aphid Transmission

**DOI:** 10.1371/journal.pone.0048177

**Published:** 2012-10-30

**Authors:** Michelle Cilia, Kari A. Peter, Michael S. Bereman, Kevin Howe, Tara Fish, Dawn Smith, Fredrick Gildow, Michael J. MacCoss, Theodore W. Thannhauser, Stewart M. Gray

**Affiliations:** 1 Robert W. Holley Center for Agriculture and Health, United States Department of Agriculture-Agricultural Research Service, Ithaca, New York, United States of America; 2 Department of Plant Pathology and Plant-Microbe Biology, Cornell University, Ithaca, New York, United States of America; 3 Department of Plant Pathology, Pennsylvania State University, University Park, Pennsylvania, United States of America; 4 Department of Genome Sciences, University of Washington, Seattle, Washington, United States of America; University of Pittsburgh, United States of America

## Abstract

Circulative transmission of viruses in the *Luteoviridae*, such as cereal yellow dwarf virus (CYDV), requires a series of precisely orchestrated interactions between virus, plant, and aphid proteins. Natural selection has favored these viruses to be retained in the phloem to facilitate acquisition and transmission by aphids. We show that treatment of infected oat tissue homogenate with sodium sulfite reduces transmission of the purified virus by aphids. Transmission electron microscopy data indicated no gross change in virion morphology due to treatments. However, treated virions were not acquired by aphids through the hindgut epithelial cells and were not transmitted when injected directly into the hemocoel. Analysis of virus preparations using nanoflow liquid chromatography coupled to tandem mass spectrometry revealed a number of host plant proteins co-purifying with viruses, some of which were lost following sodium sulfite treatment. Using targeted mass spectrometry, we show data suggesting that several of the virus-associated host plant proteins accumulated to higher levels in aphids that were fed on CYDV-infected plants compared to healthy plants. We propose two hypotheses to explain these observations, and these are not mutually exclusive: (a) that sodium sulfite treatment disrupts critical virion-host protein interactions required for aphid transmission, or (b) that host infection with CYDV modulates phloem protein expression in a way that is favorable for virus uptake by aphids. Importantly, the genes coding for the plant proteins associated with virus may be examined as targets in breeding cereal crops for new modes of virus resistance that disrupt phloem-virus or aphid-virus interactions.

## Introduction

Virus species in the *Luteoviridae*, including *Cereal yellow dwarf virus* (CYDV)-RPV, referred to herein as luteovirids, are each exclusively transmitted by one or a few species of aphids in a persistent, circulative, non-propagative manner. Aphids can transmit acquired virus for days or weeks and even after molting [1], [2], [3]. Virus is acquired when the virus moves from the aphid hindgut (HG) lumen to the hemolymph. Once in the hemolymph, the virus passes into the accessory salivary gland (ASG) and is released into the salivary duct. Aphids transmit the virus into phloem cells during salivation and feeding. Through specific interactions with putative cellular receptors [4], [5], [6], luteovirids actively cross the membranes of the aphid HG and ASG. The HG and the ASG are the major barriers to the successful aphid acquisition and transmission of luteovirids. The movement of luteovirids across these barriers is under genetic control by an unlinked set of aphid genes [7] that are additive in effect [8]. These genes encode for proteins that are differentially expressed in aphids with varying virus transmission efficiency [9], [10].

The virus encodes two structural proteins that make up the capsid. These proteins orchestrate systemic movement within host plants and transcytosis within aphids [11], [12], [13], [14]. The 22–24 kDa coat protein (CP) expressed from the viral open reading frame (ORF) 3 is the major capsid protein. Occasionally, read-through translation of the CP stop codon results in the translational fusion of ORF3 with ORF5, resulting in the minor protein species component of the capsid called the readthrough protein (RTP). Virions can be assembled from the CP alone, but the RTP is required for luteovirid movement across the ASG as well as efficient systemic infection [13], [14], [15], [16], [17]. These activities in aphids and plants are regulated by virion topological features, specifically interactions between CP monomers and well-defined interfacial regions within the RTP [18].

Although a number of aphid proteins that bind luteovirids have been identified, the molecular details of virus movement in aphids are not well understood. Due to the paucity of molecular tools for the study of aphids, most virus-binding proteins have been identified using proteomics approaches. In *Sitobion avenae*, four 2-D gel spots were differentially expressed between parthenogenetically-reproducing F1 clones that differed in their vectoring capacity for two isolates of the species *Barley yellow dwarf virus* (BYDV)-PAV [19]. Two proteins from head tissues of *S. avenae* were identified as potential receptors for BYDV-MAV based on virus overlay and 2-D immunoblot assays [5]. Other studies progressed beyond 2-D gel spot patterns to use mass spectrometry (MS) to investigate the identity of aphid proteins associated with virus transmission [9], [10], [20] in effort to link the protein function to vector biology. Membrane-associated actin, rack-1, and GAPDH-3 were identified to interact with a wild-type isolate of *Beet western yellows virus* (BWYV). Actin and GAPDH-3, but not rack-1, also interacted with two BWYV RTP mutants displaying a reduction in aphid transmission ability [20] indicating either (a) their assay for virus binding does not reflect the *in vivo* functions of these interactions in aphids, or (b), that these RTP mutants still retain the interaction interface for binding with these aphid proteins. Cyclophilin and a luciferase homologue were identified from the greenbug aphid *Schizaphis graminum* as binding to CYDV-RPV. These proteins were also differentially expressed among different *S. graminum* F2 genotypes with different CYDV-RPV vectoring capacity [9], [10]. Intriguingly, all studies investigating proteomic differences of aphid F1 [19] or F2 progeny genotypes [9], [10], [19] reported the differences between vectors and non-vectors were not related to gross changes in protein expression levels. Instead, vectoring capacity was associated with a small shift in the isoelectric point of only a small number of proteins, indicating that the control of luteovirid transmission in multiple aphid species was via the expression of allelic variants of proteins that either differentially bind to the virion or participate in the various steps of the virus transmission pathways.

In plants, luteovirids move from cell-to-cell and long distance as virions. However, in plants luteovirids must also replicate, assemble into virions, thwart the plant immune response and exhibit phloem-tissue tropism for host-to-host transmission by aphids, processes which likely involve the recruitment of a wide range of host factors to accomplish [21]. Interestingly, Bencharki and colleagues showed that the addition of soluble proteins to the aphid diet, including phloem-specific proteins that can interact with virions, can enhance transmission efficiency of the polerovirus, cucurbit aphid borne yellows virus [22]. Thus, we hypothesize that plant host proteins are also involved in aphid transmission. Serendipitously, we discovered that common additives to the plant tissue homogenization buffer during virus purification do not appear to alter the virus morphology or protein structure, but do render the virus non-transmissible by aphids. By comparing the host plant proteomes associated with transmissible and non-transmissible virions, proteins with potential involvement in transmission were identified. Moreover, our data indicate an increased accumulation of several virus-associated host proteins within aphids that have fed on infected plants, supporting a role for these host proteins in the circulative transmission process.

## Results

### Sodium Sulfite Treatment Blocks CYDV-RPV Transmission by Aphids

**Figure 1:**
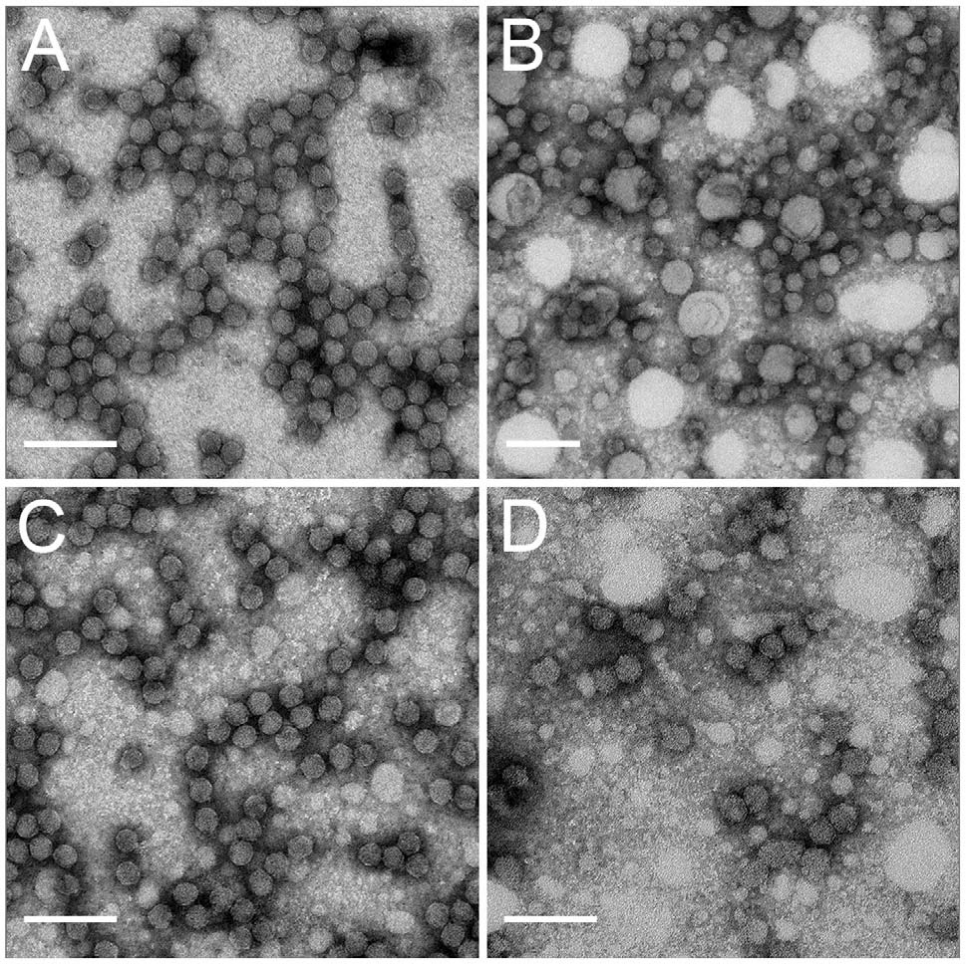
Negative stained grids, coated with CYDV-RPV coat protein antibody, of purified virus from each virus preparation after purification and virus recovered from membranes fed on by *R. padi* for a 24 h AAP. Virion morphology was similar within each group and a representative picture for each is shown. Transmissible virions after purification (A) and after membrane feeding (B) look morphologically similar. Non-transmissible virions after purification (C) and after membrane feeding (D) look morphologically similar to each other and are indistinguishable from transmissible virions in shape and size. Scale bars = 100 nm.

Sodium sulfite (Na_2_SO_3_) and ethylenediaminetetraacetic acid (EDTA) are commonly added to infected plant homogenate during virus purification to minimize the effects of polyphenolic compounds and virion aggregation, respectively [23], [24]. When sodium sulfite and EDTA were added to the tissue homogenization buffer, and the subsequent purified CYDV-RPV virions were fed to aphids through Parafilm® membranes, the virus was not transmitted in two independent experiments (Table 1). In contrast, CYDV-RPV purified without these additives was highly transmissible by aphids (>95%) in six independent experiments (Table 1). To determine whether treated virions were unstable and being degraded during membrane feeding by aphids, treated and non-treated virions were collected from the diet following the acquisition access period (AAP) and analyzed by transmission electron microscopy (TEM). Importantly, treated virions remained intact in the diet following the AAP (Fig. 1). Treated and untreated virions were morphologically indistinguishable at this point (Fig. 1). Broken capsids and capsid swelling were not observed, indicating virions were structurally stable for transmission (Fig. 1).

**Table 1:** Effects of EDTA and sodium sulfite on *R. padi* transmission of purified cereal yellow dwarf virus*-*RPV using membrane feeding assays.

	Membrane Feeding Experiment^a^						
Infected Plant Homogenate Treatment^b^	1	2	3	4	5	6	Total
**Buffer only**	18/20	12/12	16/16	16/16	11/12	11/12	84/88
**Buffer + EDTA + Na_2_SO_3_**	0/20	0/12	–	–	–	–	0/32
**Buffer + EDTA**	–	–	14/16	11/12	12/12	11/12	48/52
**Buffer + Na_2_SO_3_**	–	–	0/24	0/16	2/12	1/12	3/64

a Aphids were fed on membrane sachets containing purified luteovirus. For each experiment, the concentration of virus the same. Among different experiments, virus concentration varied from 20–60 µg/ml. Six experiments were performed using no additives, 2 experiments were performed using both sodium sulfite and EDTA, and 4 experiments were performed using only EDTA or sodium sulfite.

b For each treatment, the numbers represent total number of plants infected/total number of plants inoculated. Five aphids previously fed on membrane sachets with respective virus preparations were placed on each healthy plant.

To determine whether sodium sulfite or EDTA was responsible for the reduction in transmission efficiency, virions were purified with only EDTA or sodium sulfite as additives and transmissibility was determined. When only EDTA was added, the purified virus was transmissible at levels similar to virus prepared with neither of the additives, 92% compared to 95%, respectively (Table 1). In contrast, the sodium sulfite treatment reduced transmissibility to <5%. The negative impact of sodium sulfite on virus transmission by aphids was independent of virus concentration since the results were consistent across four independent experiments where the virus concentration in the diet varied 3-fold among experiments (Table 1).

### Sodium Sulfite Treatment of Infected Plant Homogenate Prevents CVDV-RPV Acquisition by Aphids

**Figure 2:**
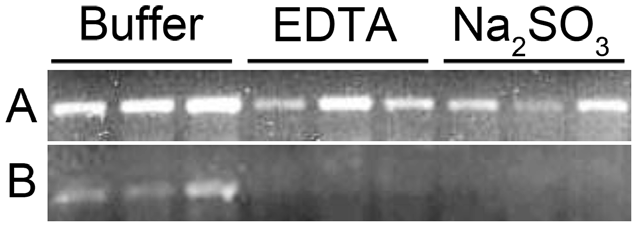
RT-PCR of total RNA extracted from *R. padi* aphids that fed on different virus preparations was performed to detect RPV using RPV coat protein primers, amplifying a 614 bp product. Aphids were collected after initially feeding for a 24 h AAP (A) and after 3 d IAP (B). Three independent replicates using RNA collected from small pools of aphids are shown for each treatment.

Transmission of CYDV-RPV is dependent upon the virus circulating through the aphid. The interaction between the virions from each purification treatment and the aphid was evaluated, particularly to identify possible barriers to transmission. To determine if aphids ingested and acquired virus, aphids were collected following a 24-h AAP on membranes containing each type of purified virus: buffer only, EDTA-treated, or sodium-sulfite treated. Total RNA was extracted from half of the aphids from each treatment and the remaining aphids were transferred to healthy oat seedlings for a 3-day inoculation access period (IAP). Total RNA was also extracted from those aphids immediately following the 3-day IAP. Viral RNA (vRNA) was detected within aphids after a 24-h AAP for each virus treatment preparation (Fig. 2) using RT-PCR and CP-specific primers. After 24-h, vRNA can be detected in all three groups of aphids. At 24-h, virus could be in the gut lumen, and/or in the HG cells and possibly in the hemolymph. This indicates aphids were ingesting, and possibly acquiring, the virus under all conditions. For aphids collected after a 3-day IAP, vRNA was detected for the buffer only treatment (Fig. 2) indicating uptake of the virus into the hemolymph, which was supported by high level of transmission (92% transmission, Table 2). No vRNA was detected after 3 day IAP (Fig. 2) for EDTA- or sodium sulfite- treated virions in the aphids. EDTA-treated virions were still transmitted by aphids to oat seedlings (92% transmission efficiency, Table 2), suggesting there was virus uptake and persistence occurred within the aphid, albeit at levels not sensitive enough to detect using RT-PCR. However, the sodium sulfite treatment reduced the rate of virus transmission into oat seedlings (Tables 1 and 2). Taken together with the RT-PCR results, these data suggest that sodium sulfite-treated virions may not be acquired. If the virus is not acquired, any virus remaining in the lumen after 24-h may flow out of the aphid in the honeydew [25].

**Table 2:** Summary of *R. padi* transmission and virion detection following membrane feeding of different cereal yellow dwarf virus*-*RPV preparations used for RT-PCR and TEM evaluations.

	RT-PCR			TEM	
	Transmission^a^	Gel data^b^		Transmission^a^	TEM observations^c^
		24 h AAP	3 d IAP		
**Buffer only**	11/12 (92%)	6/6	6/6	11/12 (92%)	5/5
**Buffer + EDTA**	11/12 (92%)	6/6	0/6	12/12 (100%)	5/5
**Buffer + Na_2_SO_3_**	1/12 (8%)	6/6	0/6	2/12 (17%)	0/5

a Purified virus that was used for RT-PCR and TEM analysis was tested for transmissibility using aphid transmission assays. Number of plants infected/total number of plants inoculated; total percent infection indicated.

b Number of aphids with detectable amount of viral RNA/total number of aphids tested.

c Number of aphids with virus in the HG cell/total number of aphids evaluated.

**Figure 3:**
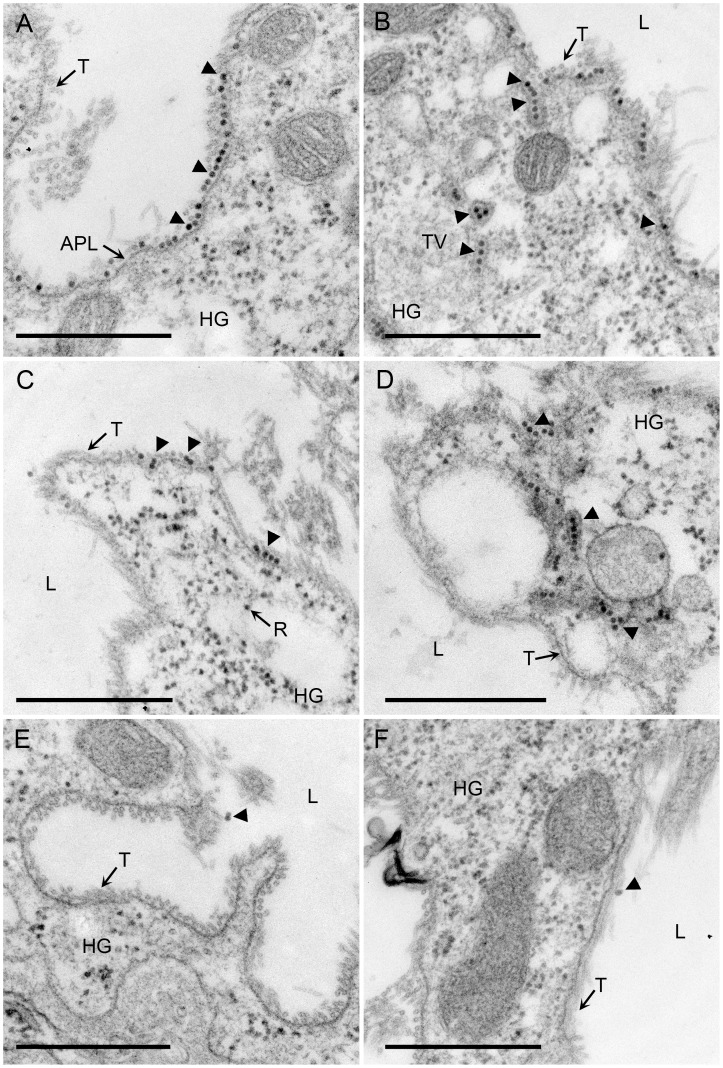
Effect of sodium sulfite and EDTA on CYDV-RPV virion (arrowhead) attachment to apical plasmalemma and endocytosis into the HG cells of *R. padi* following membrane acquisition. (A and B) no sulfite or EDTA (buffer only) virus is internalized into cells of *R. padi* HG and can be found in tubular vesicles; (C and D) EDTA only treatment shows no effect of EDTA on acquisition of virions into the aphid HG which is consistent with the transmission data presented in Tables 1 & 2; (E-F) sodium sulfite treatment prevents attachment and acquisition of virus into cells of the HG. APL, apical plasmalemma; HG, HG; L, lumen; TV, tubular vesicle; T, tubule; R, ribosome.

To determine if either additive affected the ability of virus to move into gut cells, we used TEM to visualize virion attachment and virus penetration across the gut membrane (Fig. 3). Aphids were fed on sodium sulfite- or EDTA-treated as well as non-treated virions for a 48-hr AAP. Non-treated (Fig. 3A, B) and EDTA-treated virions (Fig. 3C, D) were observed to attach to apical plasmalemma lining the HG lumen, in coated pits invaginating and budding into the cytoplasm, as well as retained in vesicles dispersed throughout the cytoplasm. In contrast, sodium sulfite-treated virions were rarely observed to interact with the apical plasmalemma nor were they observed within the cytoplasm of the HG. However, virions were observed in the HG lumen (Fig. 3E, F). The patterns of virus transmission and virus distribution within the aphid for each purified virus (non-treated, EDTA- or sodium sulfite-treated) were consistent in five aphids fed on the different virus preparations (Table 2).

After virus egress from the HG, virions must remain stable in the hemolymph and pass through the ASG for transmission to occur. To determine whether the treatments had an effect on these latter steps in the circulative transmission pathway, treated and non-treated virions were microinjected into aphid hemolymph to bypass the HG barrier. Transmission efficiency was reduced when aphids were injected with sodium sulfite-treated or sodium sulfite plus EDTA-treated virions, 8% and 0%, respectively, relative to aphids microinjected with EDTA-treated or untreated virions, 78% and 91%, respectively (Table 3). These data were consistent over multiple independent experiments, indicating the effect was reproducible and not dependent on the concentration of virus. Initial results from the membrane-feeding experiments indicated that the EDTA and sodium sulfite could be acting additively or synergistically to block CYDV-RPV transmission since no transmission was observed. An identical trend was observed in the microinjection experiments (Table 3). We performed a log-linear analysis of a 3-way contingency table using sodium sulfite, EDTA, and transmissibility as the three variables. We were unable to conclusively show any interaction effects of EDTA and sodium sulfite on aphid transmission of CYDV-RPV with the number of replicates performed for either the feeding or microinjection assays.

**Table 3:** Effects of EDTA and sodium sulfite on *R. padi* transmission of purified cereal yellow dwarf virus*-*RPV using microinjection into the aphid hemolymph.

	Microinjection Experiment^a^					
Infected Plant Homogenate Treatment^b^	1	2	3	4	5	Total
**Buffer only**	32/36	10/12	12/12	11/12	6/8	71/80
**Buffer + EDTA + Na_2_SO_3_**	0/32	0/20	–	–	–	0/52
**Buffer + EDTA**	–	–	11/12	8/12	9/12	28/36
**Buffer + Na_2_SO_3_**	–	–	2/12	0/12	1/12	3/36

a Aphids were microinjected with purified luteovirus. For each experiment, the concentration of virus the same. Among different experiments, virus concentration varied from 20–60 µg/ml. Five experiments were performed using no additives, 2 experiments were performed using both sodium sulfite and EDTA, and 3 experiments were performed using only EDTA or sodium sulfite.

b For each treatment, the numbers represent total number of plants infected/total number of plants inoculated. Three aphids previously microinjected with virus were placed on each healthy plant.

### Host-virus Interactomes are Changed by Chemical Additives to the Homogenization Buffer

To test the hypothesis that sodium sulfite treatment changes the host-virus interactome, LC-MS/MS was used to analyze tryptic digests of non-transmissible and transmissible virion preparations purified from infected oat plants. Although the genome of oat is not available, we were able to use predicted proteins from available genomes of related cereals for homology-based protein identification of the plant proteins found in the virus preparations. Twenty proteins were identified in the transmissible, but not the non-transmissible virus preparations (Table 4). Sixteen proteins (Table 4) are candidate proteins that may interact with transmissible virus directly or in complex. These include three chloroplast proteins (326533372, 20302473, and 2565305), seven mitochondrial proteins (115472339, 115474041, 115471693, 326500100, 115477529, 115448577, and 357139868), two isoforms of remorin (357164942 and 115456099), one predicted cytoplasmic protein (115449199) and three proteins with predicted functions in the nucleus (357144283, 357121487, and 226531758). Six host proteins were found in both transmissible and non-transmissible virus preparations. These include a 33-kD secretory protein, thaumatin-like protein 5 (TLP-5), ATP synthase CF1 beta subunit, cellulose synthase, triosephosphate isomerase (TIM), and a cysteine-rich repeat secretory protein (Table 4). Two proteins, prophobilinogen synthase and adenosylhomocysteinase, were identified exclusively from preparations of sodium-sulfite treated, non-transmissible virions. Proteins homologous to most of these proteins are involved in plant defense and have been previously identified in plant phloem sap proteomes [26], [27], [28], [29]. Among the potential virus genome-derived protein products, peptides from only the two virus structural proteins (CP and RTP) were identified (not shown).

**Table 4:** LC-MS/MS analysis of host plant proteins interacting with purified cereal yellow dwarf virus*-*RPV following an in solution trypsin digestion of purified viruses.

Accession^a^	Protein name	Homology^b^	Species^c^	Score	Peptides^d^	% Coverage
***Host proteins found in all virus preparations***						
gi|15239000	33-kD secretory protein		*Arabidopsis thaliana*	73.6	1	3.8
gi|326508997	Thaumatin-like protein 5		*Hordeum vulgare*	63.6	2	5.5
gi|11466794	ATP synthase CF1 beta subunit		*Oryza sativa Japonica*	69.4	6	11
gi|224075617	cellulose synthase		*Populus trichocarpa*	63.4	1	1
gi|326496613	Predicted protein	Triosephosphate isomerase	*Hordeum vulgare*	85.1	2	4
gi|357120115	cysteine-rich repeat secretory protein 55-like		*Brachypodium distachyon*	71.5	2	8.4
***Host proteins found in transmissible virus preparations***						
gi|326499075	predicted protein	Ribosomal L7Ae	*Hordeum vulgare*	78.4	2	8.8
gi|20302473	ferredoxin-NADP(H) oxidoreductase		*Triticum aestivum*	95.5	6	20
gi|115471693	dihydrolipoamide acetyltransferase E2 component of pyruvate dehydrogenase		*Oryza sativa Japonica*	71.6	4	9
gi|357144283	structural maintenance of chromosomes protein 1A-like		*Brachypodium distachyon*	93	4	4.2
gi|326500100	Predicted protein	glyceraldehyde-3-phosphate dehydrogenase A	*Hordeum vulgare*	73.8	1	4.4
gi|326533372	Predicted protein	Transketolase C	*Hordeum vulgare*	75.9	2	3.4
gi|357121487	structural maintenance of chromosomes protein 3-like		*Brachypodium distachyon*	85.9	2	2.3
gi|326500076	Predicted protein	40S ribosomal protein S9	*Hordeum vulgare*	64.6	1	9.5
gi|326496098	Predicted protein	Ribosomal protein L35Ae	*Hordeum vulgare*	61	1	9.8
gi|115477529	Os08g0536000	pyruvate dehydrogenase E1 component subunit beta	*Oryza sativa Japonica*	76.9	2	5.6
gi|297598102	Os01g0894300	Fructokinase	*Oryza sativa Japonica*	63.5	2	6.5
gi|115474041	Os07g0675000	Acyl-CoA dehydrogenase	*Oryza sativa Japonica*	94.4	3	4
gi|115456099	Os03g0808300	Remorin C	*Oryza sativa Japonica*	68	2	8.5
gi|115448577	putative pyruvate dehydrogenase E1 component alpha subunit		*Oryza sativa Japonica*	75.2	2	3.0
gi|226531758	hypothetical protein LOC100279994	Nucleosome assembly protein	*Zea mays*	65.1	2	6.1
gi|357139868	dihydrolipoyllysine-residue acetyltransferase component 1 of pyruvate dehydrogenase complex		*Brachypodium distachyon*	65.1	3	2.8
gi|357164942	remorin-like isoform 1		*Brachypodium distachyon*	74.8	2	9.6
gi|2565305	glycine decarboxylase P subunit		*Tritordeum sp*	80	2	2.7
gi|115472339	Os07g0513000	ATP synthase gamma chain	*Oryza sativa Japonica*	59.5	2	6.4
gi|115449199	Os02g0794700	Cytosol aminopeptidase family	*Oryza sativa Japonica*	75.5	2	3.7
***Host proteins found only in non-transmissible virus preparations***						
gi|115469822	Os06g0704600	porphobilinogen synthase	*Oryza sativa Japonica*	48.3	2	6
gi|297804932	Hypothetical protein	Adenosyl-homocysteinase	*Arabidopsis lyrata*	72.3	2	5

a NCBI accession number as of Feb. 20, 2012.

b If the gene was not annotated, functional homology to an annotated gene was determined using BLASTP [92].

c Protein identification was achieved using homology-based searching in MASCOT [93] because the genome of *Avena sativa* (oat) is not available. The species of the top MS/MS match is reported.

d Number of unique peptides with different sequences matched to a homologous protein with a peptide identification probability >95% as specified by the Peptide Prophet algorithm [90]. Each unique peptide match is based on at least 2 distinct spectra, but in some cases, many more. In the case of cysteine-rich repeat secretory protein 55-like, more than 300 total spectra were matched, highlighting the limitations of a homology-based search strategy for protein identification [79].

To rule out co-sedimentation of plant proteins with purified virus based on density alone, we used LC-MS/MS to thoroughly characterize sucrose gradient fractions of healthy plant homogenates (Table S1). Healthy plants were grown in the greenhouse alongside infected plants and the tissue was used in the virus purification protocol. Fractions from the gradient that would normally contain virus particles when infected plant tissue is used in the purification were subjected to trypsin digestion and LC-MS/MS to identify any plant proteins that could co-sediment with virus particles based on density and not a physical association. Four proteins (Table 4) that are likely not associated with virions, including homologues of three ribosomal proteins and fructokinase, were also identified in the analysis of the gradient fractions from healthy plants. Other abundant proteins detected in the sucrose gradients from healthy tissue included Rubisco, pyrophosphate-dependent phosphofructo-1-kinase, 6-phosphofructokinase, ribosomal proteins, and histone proteins (Table S1). Although many of these proteins have been detected in phloem sap in other plant species [26] and in the gradients of purified virus, the presence of these proteins is probably unrelated to the transmissibility of virions since they were detected in the fractions from healthy tissue. Importantly, no peptides were identified as belonging to CYDV-RPV indicating that the plants used for the healthy controls were not infected.

Analysis of purified sodium sulfite and EDTA-treated and non-treated CYDV-RPV using 1-D gel electrophoresis often showed distinct protein profiles (Fig. S1A). These differences were initially thought to be due to degradation products of the RTP; since the C-terminal half of the RTP is truncated during purification [30], [31]. For both transmissible and non-transmissible virus, we analyzed gel bands corresponding to viral and host proteins. MS analysis of peptides recovered from an in-gel trypsin digestion of band containing the truncated RTP from purified virions produced nearly identical tryptic fragments representing both the CP and RTP from both treated (Fig. S1B) and untreated virions (Fig. S1C). These data indicate minimal or no differences in the virus protein composition of the virion particles. Consistent with previous reports of C-terminal truncation of the RTP during purification, no peptides were discovered in the C-terminal portion of the RTP from either type of purified virus. The highly abundant proteins (Table S1) were also found in analysis of the gel bands from Fig. S1 (data not shown) and enabled us to attribute some of the variability in the gel bands to differences in plant protein complexes that co-sediment based on their density alone.

### CYDV-RPV-associated Host Proteins Accumulate Differentially in Aphids Fed on Infected Plants

The LC-MS/MS data suggest that treatment of plant tissue homogenate with sodium sulfite disrupts the host-virus interactome that is required for virus transmission by aphids. Here, the host-virus interactome is defined as the complement of host plant proteins binding directly to or in complex with the purified virus. The host proteins may play a direct role in virus uptake, maintaining virion stability, or help to provide enzymatic or co-factor activity during initial steps of entry into aphid cells. We hypothesized that if host plant proteins were involved in transmission, either via direct interactions with virions or by another indirect mechanism, we should be able to detect evidence of these proteins accumulating to higher levels in aphids that have fed on infected plants compared to aphids that have fed on healthy plants. A targeted proteomics approach called selective reaction monitoring (SRM) mass spectrometry [32], [33], [34], [35] was used to detect peptides derived from virus-interacting plant proteins in aphid protein homogenates. SRM detects and quantifies selected tryptic peptides within a total protein extract that are unique to the proteins of interest by monitoring specific intact tryptic peptides ions and their collisionally-induced dissociation (CID) fragments (ions derived from fragments of the tryptic peptides) based on *in silico* predictions of their mass:charge ratios. In contrast to the discovery approach that enabled us to ask “What host proteins are in the different virus preparations?” (Table 4), SRM enables us to ask the hypothesis-driven question, “Are virus-interacting plant proteins A, B, and C, present in our aphid protein sample?”.

The candidate host proteins considered for SRM studies included all of the proteins listed in Table 4 that were found to be exclusively associated with transmissible virus. Proteins found in both transmissible and non-transmissible virus were also considered since they may be found in different amounts in the aphid. All of these plant proteins were checked for similarity against all available aphid and aphid bacterial endosymbiont protein sequences. Peptides from proteins that were identical to aphid or endosymbiont proteins at the amino acid level were not considered for further analysis as only peptides unique to the plant proteins could be informative for the specific measurements of the plant proteins in aphids. Those that remained included cellulose synthase, thaumatin-like protein 5, 33-kD secretory protein, pyruvate dehydrogenase E1, pyruvate dehydrogenase E2, remorin, cysteine-rich repeat secretory protein 55-like, structural maintenance of chromosomes protein 3, and predicted ribosomal protein. We also included β-D-glucosidase which was abundant in healthy controls as well as the purified virus preparations. To perform relative quantification of these proteins, a method was created in Skyline [32] that identified peptides specific to each of the candidate virus-interacting host proteins and exported to a TSQ Vantage mass spectrometer operating in SRM mode. Total protein extracted from the efficient RPV-vector aphid species *Rhopalosiphum padi* (all developmental stages) fed on healthy or infected plants for 21 days was digested with trypsin and analyzed by SRM. Peptides from five of the plant proteins listed in Table 4 were detected in the *R. padi* homogenate (Table 5). A new method was created containing only these peptides, and three biological replicate samples of digested homogenates from aphids reared on infected or healthy plants were analyzed. The chromatographic retention time for each peptide was highly reproducible among all six samples (Table 5). The transitions are generally free from interference when they were detected (Fig. S2, Table S2) in the digested homogenates that were derived from aphids fed on infected plant tissue. Only one peptide from thaumatin-like protein 5, 33-kD secretory protein and pyruvate dehydrogenase E1 and E2 was detected (Table 5), whereas two peptides from cellulase were detected (Table 5, Figure S2). Until the oat genome is sequenced, we do not know exactly what protein we are monitoring, i.e., a single plant protein isoform or a mixture of proteins sharing the same peptide.

**Table 5:** Peptides from cereal yellow dwarf virus-RPV-interacting host proteins detected inside *R. padi* using selected reaction monitoring mass spectrometry.

				Chromatographic retention time^c^					
				RPV+			Healthy		
Accession number^a^	Protein name	Fold-change^b^	Peptide	1	2	3	1	2	3
gi|224075617	cellulose synthase	−0.3	SQTGDFDHNR	21.1	19.6	20.6	21.9	23.4	25.7
		0	IPMFAYVSR	39.9	39.7	40.1	40.3	41.0	41.5
gi|56682582	Thaumatin-like protein 5	+2.0	FGGDTYCCR	5.3	5.2	5.3	5.6	5.7	6.2
gi|15239000	33-kD secretory protein	+1.6	VLYSSCYVR	25.4	25.5	26	26.1	26.7	ND
gi|115448577	pyruvate dehydrogenase E1component alpha subunit	+2.6	SDSIITAYR	7.1	7	8.2	7.7	7.7	8.2
gi|115471693	dihydrolipoamide acetyltransferase E2component of pyruvatedehydrogenase	NC	GLGMIAEEVK	6.4	6.4	6.7	ND	ND	ND

a NCBI accession number as of Feb. 20, 2012.

b Fold-change reported is a log2(fold-change) for the peptide. Fold-change is therefore >0 for proteins that are found more abundantly in aphids fed on infected tissue and <0 for proteins that accumulate less abundantly in aphids fed on infected tissue, p<0.05 except for gi|224075617, which was not differentially detected within aphids between the treatments. NC indicates we did not calculate a fold-change.

c Average time (in minutes) peaks were found to elute off the column in three replicates of aphids fed on infected (RPV+) or healthy (RPV-) tissue. ND indicates that we did not detect the transitions in that sample.

Total peak areas for the selected peptide ions and their CID fragments were used to calculate a fold-change, and the log [2] of the fold-change is reported so that a positive value indicates an enrichment of the peptide in the extract from aphids fed on CYDV-RPV infected plants, and a negative value indicates a lower level of the peptide in the extract from aphids fed on CYDV-RPV infected plants. Pyruvate dehydrogenase E1 and E2 were found to associate with only transmissible virions (Table 4). Peptides derived from both of these proteins were found to accumulate to higher levels in aphids fed on CYDV-RPV infected plant tissue (Table 5). In fact, the peptide from pyruvate dehydrogenase E2, GLGMIAEEVK, was only detected in aphids fed on infected plant tissue (Table 5) and not in aphids fed on healthy plants. Cellulose synthase, thaumatin-like protein, and the 33-kD secretory protein co-purified with both transmissible and non-transmissible virions (Table 4). Cellulose synthase was detected at similar levels in aphids reared on healthy and infected plants, with fold-changes consistent across two different peptides from this protein (−0.3 and 0). In contrast, peptides specific to thaumatin-like protein 5 and 33-kD secretory protein were found to accumulate to higher levels in aphids that had fed on infected plants (Table 5). These data suggest that the amount of these proteins was increased in aphids fed on CYDV-RPV infected tissue, with fold-changes of 2.0 and 1.6, respectively. Peptides from remorin, cysteine-rich repeat secretory protein 55-like, structural maintenance of chromosomes protein 3, predicted ribosomal protein, and β-D-glucosidase were not detected. SRM is excellent at reproducibly monitoring peptide signals; however, upon finding interesting differences such as the ones described above, the next step is validation and absolute quantification via the use of stable isotope labeled peptides.

## Discussion

Successful virus purification depends on obtaining high titers of virus in host plants and having methods to efficiently extract biologically active virus from infected tissue. The latter is most critical since virus particles must retain their infectivity while facing the harsh oxidative environment during plant tissue homogenization. Ideally, purification methods should be optimized to obtain a high titer while maintaining biological activity for each virus species under investigation. Purified BYDV (later to be recognized as BYDV-MAV) from Coast black oats was first reported by Rochow and Brakke [36], using 0.1 M pH 7 phosphate buffer with no additives included in the homogenization buffer. Pierpoint [37] identified a number of substances (ascorbate, ethyl xanthate diethyldithiocarbamate, cysteine, 2-mercaptobenzothiazole) that prevented oxidation of polyphenolic compounds in homogenized, infected plant tissue (which can be observed as browning of the plant homogenate). As a result, reducing agents became commonplace additives to virus extraction buffers and, in many cases, with positive outcomes. In contrast to our current study, the addition of sodium sulfite and EDTA were necessary for successful purification of the potyvirus, peanut mottle virus [38]. When purifying BYDV-PAV, Hammond (1983) incorporated sodium sulfite in the extraction buffer to prevent browning, and its addition did not affect infectivity nor did reducing agents affect other luteovirids such as the potato leafroll virus (PLRV) [14] or soybean dwarf virus (B. Tian, personal communication). However these studies did not examine the virus transmissibility in the absence of reducing agents so it is unknown whether sodium sulfite imparts any negative (or positive) effects on the transmissibility of other purified luteovirids. Those reviewing plant virus purification protocols [24] cautiously warn against adoption of a one-size-fits-all protocol for purifying plant viruses. Together with the previous works, our data show such caution should be duly noted by those purifying luteovirids. The vector-virus specificity that defines the luteovirids undoubtedly reflects the complex chemical nature of protein interactions mediated by the CP and RTP. Care should be taken in understanding how the chemistry of the purification protocol can impart biochemical changes in the virion that will alter virus-host and virus-vector protein interactions.

The action of sulfite on disulfides (e.g. cystines) can be represented by the equilibrium equation: R.S.S.R + SO_3_^2−^ ↔ RS^−^ + RS.SO_3_^−^. Above pH 9, the equilibrium constant does not favor the total cleavage of the disulfide bond, and large excesses of sulfite are required to drive the reaction to completion. However, at lower pH values (e. g. pH 7 of the homogenization buffer used in these virus preparations) the thiol predominates over the thiolate ion resulting in more complex reaction kinetics and more favorable equilibriums [39]. In general, the equilibrium constant can be shifted to the right by any process that removes the thiol and in the presence of divalent metal ions, known to stabilize several icosahedral viruses [40], and all thiol and disulfide groups can be readily converted to S-sulfonates by a process that reduces the metal ion [41]. The reaction of sulfite with protein disulfides is further complicated by an acute sensitivity to the ionic atmosphere in the neighborhood of the disulfide bond. For example, anionic disulfides have been shown to react much more slowly than neutral or cationic disulfides [42]. For cystines (disulfides) having two positively charged flanking amino acids, the rate constant for reaction of the negative disulfide is 132,000 s^−1^M^−1^ as compared to one having two neutral neighbors, 367 s^−1^M^−1^, a 10^6^-fold range in rate constants [43]. Furthermore, the rate of reaction can be greatly affected by steric factors, and the disulfide bonds of many proteins show great variability in their susceptibility to cleavage by sulfite in the absence of denaturants such as guanidine hydrochloride or urea [44], [45], [46], [47]. Thus, it can be expected that in the absence of denaturants, the thiols and disulfides most susceptible to modification by sulfite would be those that are most exposed and that have positively charged amino acids as their nearest neighbors.

With these thoughts in mind, the three cysteines in the CP or RTP of CYDV-RPV may provide some clues (Fig. S2) as to why sodium sulfite treatment would affect the virus-plant interactome. Two of the cysteines are flanked by at least one basic amino acid. These are likely the most reactive cysteines in the CP or RTP with respect to the formation of S-sulfonates and would readily participate in thiol-disulfide exchange reactions initiated by the sulfolysis of other disulfide bonds. Blocking the critical thiol by converting it to a thiosulfonate or otherwise modifying critical interactions via the formation of a non-native disulfide could have negative consequences for the internalization of RPV into aphid cells, as is observed for a wide range of other viruses (enveloped and non-enveloped) that rely on disulfide bond formation for internalization into and transport through host cells [48], [49], [50]. In contrast to the sodium sulfite treatment, EDTA treatment did not prevent entry of virus into aphid cells or transmissibility, but it did appear to reduce the long term stability of the virus inside the aphid. Hence, *in vivo* interactions between divalent cations and the virus capsid may be required for long-term virion stability. Indeed, other icosahedral plant viruses such as rice yellow mottle virus, tomato bushy stunt virus, southern bean mosaic virus and cowpea chlorotic mottle virus are stabilized by divalent cations such as Ca^2+^ and Mg^2+^, [40], [51], [52], [53], [54].

These data support the hypothesis that transmission of CYDV-RPV requires the formation of a critical disulfide bond pairing either intramolecular, within the CP or RTP, or intermolecular with a specific host protein, and that treatment with sodium sulfite promotes a random process of thiol-disulfide exchange that creates structures that interfere with normal virus transmission. Sodium sulfite-treated virions did not enter into the HG or into the ASG and thus, a partial overlap in the biochemical mechanisms for virus entry may exist in these two aphid tissues. Previous work using infectious mutants of other luteovirids supports the role of cysteine residues within the capsid as contributing to virus-host specificity and even aphid transmission. PLRV mutants with cysteine deletions or modification of residues flanking cysteines residues show phenotypes in a host-dependent manner Two mutants D-P-K (which alters two cysteine-flanking residues) and H-C-K (which deletes the cysteine residue) showed defects in systemic infection in the host *Physalis floridana*, but not in *Solanum tuberosom*, *Nicotiana benthamiana*, or *Nicotiana clevelandii* [11]. Aphid transmission of the D-P-K mutant was severely impaired when acquired from or inoculated into *P. floridana* [11].

LC-MS/MS enabled us to detect numerous host proteins co-purifying with virion particles in the sucrose gradients. The SRM data indicate a complex picture on the roles of these plant proteins in luteovirid transmission by aphids. How might host proteins also participate in aphid transmission? As a result of natural selection, aphid acquisition of CYDV-RPV by aphids may have been favored from phloem cells with higher protein expression, a phenotype that could have evolved as a response from virus infection, the act of aphid feeding, or both in combination. Co-ingestion of soluble plant proteins that associate with virions may help stimulate endocytosis into the epithelial cells of the aphid HG in a host-dependent manner. These data are consistent with previous observations of host proteins as associated with insect-transmitted viruses [21], [22] and that addition of any soluble protein into the diet acquired together with virus can enhance transmission efficiency [22]. In tobacco BY-2 cells, sugar levels tightly regulate pyruvate dehydrogenase E1 and E2 promoters. Promoter activity is markedly increased by sugar depravation [55]. We detected pyruvate dehydrogenase E1 and E2 at increased levels in aphids fed on CYDV-RPV infected plants. In contrast, cellulose synthase was detected at similar levels in aphids fed on healthy or CYDV-RPV infected plants. This is consistent with a more general role for the latter protein in aphid-plant interactions that CYDV-RPV may exploit. In varieties of wheat that are susceptible to the phytotoxic Russian wheat aphid, *Diuraphis noxia*, the mRNA encoding for cellulose synthase is up-regulated 4 to7-fold during aphid infestation [56]. Intriguingly though, evidence suggests that the Russian wheat aphid is a poor vector for yellow dwarf viruses [57], [58]. Cellulose synthase is a member of the glycosyltransferase family A protein family. These proteins synthesize glycoconjugates by transferring a sugar moiety to a donor molecule, such as a protein or lipid. Little is understood about the role of protein glycosylation in non-enveloped viruses – specifically plant viruses [59] and a role for glycosylation in luteovirid transmission is not well understood [59], [60].

Independent of a direct role in aphid transmission, cellulose synthase and the other virus-associated host proteins may help to orchestrate virion functions *in planta*. Most plant viruses, including CYDV-RPV, move from cell-to-cell in host plants via plasmodesmata (PD, reviewed in [61]. Luteovirids are targeted to PD early during infection [62]. PD permeability is regulated by carbohydrate metabolism via callose deposition [63]. Callose turnover regulates PD size exclusion limit, as ectopic expression of m-type thioredoxin that is expressed in non-green plastids, which controls callose deposition, causes an increase in plasmodesmal permeability [63]. For cell-to-cell movement, plant viruses have evolved specialized mechanisms to tap into the plants endogenous system for controlling PD permeability for their own cell-to-cell transport. We propose cellulose synthase may be involved in modification of the cell-wall encasing the specialized PD to assist in cell-to-cell virion translocation. A number of other plant viruses have been known to directly interact with and use host enzymes involved in carbohydrate breakdown for cell-to-cell movement through plasmodesmata [21], [64], [65]. Susceptibility to virus infection is decreased in a class I beta-1,3-glucanase-deficient mutant of tobacco generated by stable transformation of tobacco with an antisense construct. The mutant exhibited delayed intercellular trafficking via PD of a tobamovirus (tobacco mosaic virus), of a potexvirus (potato virus X), and of the movement protein 3a of a cucumovirus (cucumber mosaic virus), as well displayed alterations in callose deposition. Through interactions with host beta-1,3 glucanase, the triple gene block protein of PVX, TGB 12 modulates plasmodesmal permeability, probably to mediate cell-to-cell spread [66]. Viruses that move from cell-to-cell as ribonucleoproteins (RNPs) also recruit cell-wall modifying enzymes for cell-to-cell movement [64], suggesting that enzymatic modulation of cell wall carbohydrates to alter PD permeability is a strategy widely used by plant viruses for intercellular trafficking.

Several of the proteins found associated with CYDV-RPV have well-described functions in nuclei, for example SMC1, SMC3 [67], and NAP [68]. Luteovirids are positive-sense, single stranded RNA viruses. Although the coat protein and the RTP of the related luteovirid PLRV can localize to the nucleolar compartment of plant cell nuclei, this localization is lost in the presence of replicating vRNA [69]. Furthermore, in the early stages of infection of oats with BYDV, infected cell nuclei become morphologically distorted and filaments associated with cytoplasmic virions appear in the nucleoplasm and in nuclear pores [62]. Thus, a physical interaction between coat protein and these proteins, *in vivo*, is an intriguing possibility. However, the latter three proteins have been reported to have additional, non-nuclear functions and/or localization [70], [71], [72]. Furthermore, aphid SMC proteins are commonly found associating with purified PLRV in co-immunoprecipitation studies (Cilia, unpublished). It is also possible that maturation of phloem sieve elements may release these proteins into the sap and provide a mechanism for functional protein interactions with luteovirids, or that these proteins are transported into the phloem sap to carry out functions yet to be defined. A detailed LC-MS/MS analysis of the pumpkin phloem sap proteome revealed numerous ribosomal proteins and homologues to NAP and many other proteins with nuclear functions [26].

Excitingly, we found two remorin proteins to co-purify with transmissible virus (Table 3). Consistent with remorin localization in plant cell membranes [73], peptides derived from remorin were never detected from aphid homogenates (data not shown). Remorins are plant-specific proteins with unknown functions [74] but have been receiving wide attention because of their involvement in plant defense against viral, bacterial, and rhizobial infections [reviewed in [75]]. *In vivo*, remorins cluster in the plasma membrane within PD and lipid rafts [73]. Remorin proteins accumulate in mature and aging tissues and in source tissue [76] where mature, branched PD are in the majority. Remorin can physically interact with the PVX movement protein TRIPLE GENE BLOCK PROTEIN 1. Remorin association with PVX is inversely proportional to the ability of PVX to move from cell-to-cell [73]. These data show that remorin proteins may function in vivo to retain virus within individual cells. Remorin association with luteovirids is particularly interesting because the luteovirid RTP retains virus in the phloem. Phloem-retention of luteovirids is critical for virus dispersal by aphids [13]. How this occurs is not known, but one possibility could be via protein-protein interactions between the C-terminal domain of the RTP and remorin. Luteovirid mutants that are no longer restricted to the phloem [13] will be particularly useful to probe direct interactions with remorin proteins.

### Conclusions

There is a paucity of tools to study vector biology and vector-virus-host interactions at the molecular level; however, mass spectrometry technologies are emerging as one of the most powerful tools to develop a comprehensive understanding of virus-vector-host interactions [9], [18], [22], [77], [78], [79], [80], [81], [82], [83]. We used mass spectrometry to describe several host proteins that associate with virions and to show they may even be ingested by aphids during feeding. The mounting body of evidence is that luteovirids commandeer their hosts and vectors to ensure their own survival and transmission. For instance, luteovirids move from companion cells into sieve elements but natural selection has favored their retention in the phloem of host plants and hence, dispersal by aphids. Luteovirid infection may also exert changes in the phloem proteome, changes that may also facilitate virus dispersal by aphids. A critical next experiment would have to distinguish between the following two hypotheses a) that virion-host protein complexes are internalized into aphids or b) that the virus manipulates the phloem to have a higher concentration of these proteins during infection. Due to the dynamic nature of protein trafficking in plants [61], [63], [84], [85], [86], [87] and cell-type specific transcriptional regulation [88], the latter experiment is not a trivial undertaking. The current work has laid the groundwork for these future experiments. The virus-host interactome we describe in this study provides critical insights into the biochemical mechanisms that luteovirids use for movement in plants and aphids. These plant proteins (and associated biochemical pathways) are novel targets for developing host-resistance to luteovirid infection in cereals and other crops. Furthermore, the exciting, serendipitous discovery that sodium sulfite reduces transmissibility of virions provides biochemical evidence that intra or intermolecular disulfide bonding may be required for luteovirid entry into aphid cells and may also be exploited as part of a strategy to disrupt aphid-virus interactions and ultimately to mitigate virus transmission.

## Materials and Methods

### Virus Purification

CYDV-RPV was purified from oat plants (*Avena byzantina* K. Koch cv. Coast black) inoculated 7 to 8 weeks previously with viruliferous *R. padi* as described [10]. Infection was determined by yellowing and dwarfing symptoms. Tissue harvested was divided into 200–300 g batches, chopped into 2.5 cm pieces and frozen at −80°C until used. Virus was purified using a modified version of the protocol of Hammond et al. [89]. Tissue was homogenized using 0.1 M phosphate (K_2_HPO_4_) buffer (pH adjusted to 7 using 0.1 M KH_2_PO_4_) at 2.5 ml g^−1^ tissue. Tissue was homogenized with phosphate buffer containing 1% cellulase with and without 0.01 M EDTA and 0.5% sodium sulfite together or individually. Sucrose gradients were fractionated using a density-gradient fractionator (Teledyne-ISCO) at sensitivity 0.5 and chart speed set at 60 cm/h. Two milliliter fractions were collected along the entire gradient for each gradient. The virus fractions were collected as 4 ml gradient fractions and were concentrated by centrifugation for 4 h at 113,613×g in a type 70Ti rotor (Beckman Coulter). The remaining fractions were stored at −80°C until needed. Supernatant was discarded and the pellet was resuspended overnight in 0.01 M phosphate buffer, pH 7. Purified virus was equally divided among several tubes and stored at −80°C. As a control, 500 g of healthy tissue was homogenized in phosphate buffer containing 1% cellulase and purified following the above protocol.

### Evaluation of Purified Virions

Purified virus from each sample preparation after purification was evaluated by negative staining. In addition, virus was recovered from membranes after aphids fed for 24 h to assess the stability of the virus. 300 Mesh copper carbon-coated formvar grids were incubated for 30 min on a 10 µl drop of RPV coating antibody diluted 1∶500 in phosphate buffered saline (PBS). Excess antibody was wicked off with a wedge of filter paper and grids were rinsed in 3 drops of PBS, wicking off excess with filter paper after each rinse. Antibody-coated grids were incubated for 1 h on a 10 µl drop of virus. Virus was wicked off using filter paper and grids were rinsed in 2 drops of PBS, followed by 3 drops of sterile distilled water, wicking excess after each rinse. Grids were stained by incubating for 3 min on a 20 µl drop of 2% aqueous uranyl acetate, excess stain was wicked off and grids were stored dry in a grid box. Grids were viewed on a Jeol 1200 TEM at the Electron Microscope Facility for The Huck Institute of the Life Sciences at The Pennsylvania State University.

### Aphid Transmission Assays

For each purified virus preparation (buffer only; buffer including EDTA and sodium sulfite; buffer including EDTA only; buffer including sodium sulfite only), healthy *R. padi* aphids were allowed a 24–48 h AAP on stretched Parafilm® membranes made by standard protocol by stretching in two directions until very thin. Approximately 75 µl of the virus preparation, at 20–60 µg/ml concentration containing 15% sucrose was used in each membrane. After 24 h, aphids were allowed a 3–4 day IAP on 7 day old oat seedlings (Coast black), 5 aphids per plant, 12–16 plants per treatment. Plants were fumigated with Orthene to kill the aphids. The fumigated plants were placed in the greenhouse and observed for symptom expression 3–4 weeks later. Infected plants were evaluated by obvious yellowing and reddening of the leaves, and dwarfing of the plant. In addition, a randomly selected subset of the leaves (symptomatic and asymptomatic) was tested by double-antibody sandwich enzyme linked immunosorbent assay (DAS-ELISA) using anti-CYDV-RPV antibodies. Each type of virus preparation was tested 2–4 times.

In addition to membrane feeding, virus was directly injected into the hemocoel of the aphid to bypass the HG. For each purified virus preparation, 10 nl of a 20–60 µg/ml virus preparation was injected into an aphid using a Microinjector IM300 (Narishige). Three aphids were placed onto a 7 day old oat seedling (Coast black), 12–16 plants per virus treatment, for a 4 day IAP, after which plants were fumigated to terminate feeding. Plants were evaluated for infection 3–4 weeks later as described above. To test for synergistic effects of EDTA and sodium sulfite treatment on CYDV-RPV transmission by *R. padi*, we performed a log-linear analysis for a three-way contingency table on the transmission results following microinjection using +/− EDTA, +/− sodium sulfite and +/− transmission as the three variables. Simulations and the experiment were run using the statistical software package JMP (SAS).

### Virus Detection in Aphids

Healthy *R. padi* aphids were allowed a 24 h AAP on membranes, as described previously, which contained 40 µg/ml of purified virus in 15% sucrose buffered with 0.01 M phosphate buffer, pH 7. The following virus treatments used were preparations containing 1) phosphate buffer only, 2) phosphate buffer including EDTA only, and 3) phosphate buffer including Na_2_SO_3_ only. To examine an aphid’s ability to acquire virus, after 24 h, six aphids were collected, divided randomly into three groups of two for each treatment and stored at −80°C in 25 µl nuclease-free water in an RNase-free microcentrifuge tube. The remaining aphids were transferred to healthy oat seedlings (Coast black), placing five aphids per seedling. Aphids were allowed a 3 day IAP to test for transmissibility of the virus, and aphids were collected to examine the treatment effects on virus uptake into the hemocoel. For each virus treatment, six aphids were collected, divided randomly into three groups of two for each treatment and stored at −80°C in 25 µl nuclease-free water in an RNase-free microcentrifuge tube. Total RNA was isolated, five µl of RNA was used in an RT-PCR as described [14], with the exception that primers specific for the coat protein of CYDV-RPV (RPV-CP For: 5′-ATGAGTACGGTCGTCCTTAGATCC-3′; RPV-CP Rev: 5′-CTATTTTGGGTTTTGTAGCTGGAC-3′) were used to amplify a 614 bp fragment.

TEM was also used to detect virus within the aphid. *R. padi* were allowed a 48 h AAP on membranes containing 40 µg/ml per virus preparation. For each virus preparation, ten aphids were collected after feeding, the head and cauda were removed using a razor blade, and heads and abdomens were fixed for TEM in 1% formaldehyde-2% gluteraldehyde in 0.02 M sodium cacodylate (pH 7.2) containing 10 mM calcium chloride and 0.05% sodium azide for 24 h. Aphids were subsequently prepared for TEM accordingly [13]. The HGs of five aphids were examined per virus treatment. Grids were viewed on a Jeol 1200 transmission electron microscope (TEM) at the Electron Microscope Facility for The Huck Institute of the Life Sciences at The Pennsylvania State University.

### Electrophoresis

25 µg of virus preparations were separated on 10–20% Novex tricine gels (Invitrogen) according to the manufacturer’s instructions, at 125 V for 2 hr at room temperature in the SureLock XCell minicell (Invitrogen). Gels were fixed for 30 min in 100 ml of a solution containing 50% methanol and 7% acetic acid. After 30 min, the fixing solution was replaced with fresh fixing solution. The gels were stained overnight with a 1∶1 dilution of Sypro Ruby stain (Invitrogen) in nanopure water at room temperature in the dark. The gels were then washed for 30 min in a solution containing 10% methanol and 7% acetic acid. Gels were scanned on the Typhoon Variable Mode Imager (GE Healthcare) at 100 dpi and visualized with the 532 nm laser using the 610BP30 filter. The bands were excised from the gel using a bench top UV transilluminator at a wavelength of 302 nm.

### LC-MS/MS

#### Gel bands

Each gel band was subjected to an in gel tryptic digestion and extraction as described [77]. Dried samples were reconstituted with 12 µL of 3% acetonitrile (ACN) with 0.1% trifluoracetic acid. Nano-LC (nLC) separation of tryptic peptides was performed with a nanoACQUITY system (Waters), equipped with a Symmetry C_18_ 5 µm, 20 mm×180 µm trapping column and a UPLC BEH C_18_ 1.7 µm, 15 cm×75 µm analytical column (Waters). The samples were transferred to the trapping column using a 5 µL partial loop injection with a 0.1% solution of formic acid (FA) in water at a flow rate of 7 µL/min for 3 min. Mobile phase A consisted of 0.1% FA in water and mobile phase B consisted of 0.1% FA in ACN. Following desalting and concentration, the trapping column was subjected to a reverse flush to the analytical column and separated with a gradient of 2–40% mobile phase B over 60 min at a flow rate of 300 nL/min, followed by a 5 min rinse with 95% of mobile phase B. The column was re-equilibrated at initial conditions for 20 min. Column temperature was maintained at 35°C. 100 fmol/µL [Glu^1^]-fibrinopeptide B in 25% ACN with 0.1% FA was used as the lock mass compound and was delivered via the auxiliary pump of the LC system at a flow rate of 300 nL/min to the reference sprayer of the NanoLockSpray source of the mass spectrometer. The eluent from the analytical column was delivered to the analytical sprayer of the same source through a PicoTip emitter (New Objective, Woburn, MA) with 10 µm tip diameter.

Mass spectrometric analysis of tryptic peptides was performed using a Synapt HDMS mass spectrometer (Waters, Manchester, UK) or a 4700 Proteomics Analyzer (Applied Biosystems) as described in [78] and [77], respectively.

#### In solution digests of purified virions

For analysis of purified CYDV-RPV virions in solution, disulfide bonds were reduced with 5 mM dithiothreitol (DTT) for 30 min, followed by alkylation with 10 mM iodoacetamide (IAA) for 30 min, in the dark. Reduction and alkylation were performed at 25°C. CYDV-RPV virions were then digested with trypsin (1∶200 ratio, Promega) at 35°C for 16 h. The peptide mixture was desalted using a C18 sep-pak (Waters) and stored at −80°C for 1 week prior to MS analysis.

For nLC-MS/MS analysis on the LTQ-Orbitrap Velos (Thermo-Fisher Scientific, San Jose, CA), the tryptic digest was reconstituted in 10 µL of 2% ACN with 0.5% formic acid (FA). The mass spectrometer was equipped with a “Plug and Play” nano ion source device (CorSolutions LLC, Ithaca, NY). The nanoLC was performed using a Dionex UltiMate3000 MDLC system (Dionex, Sunnyvale, CA). The gel extracted peptides (5–10 µL) were injected using a “User Defined Program” onto a PepMap C18 trap column (5 µm, 300 µm×5 mm, Dionex) at a 20 µL/min flow rate for on-line desalting and then separated on a PepMap C18 reverse phase (RP) nano column (3 µm, 75 µm×15 cm, Dionex) which was installed in the “Plug and Play” device with a 10-µm spray emitter (NewObjective, Woburn, MA) mounted in front of the Orbitrap ion transfer tube. The peptides were then eluted in a 60 min gradient of 10% to 40% ACN in 0.1% FA at 300 nL/min. The Orbitrap Velos was operated as described previously [9]. For repeat injections of the same samples, an exclusion list containing m/z values identified in the previous DDA run were generated using Proteome Discovery 1.1 software and applied to prevent resampling of the same ions. All data were acquired using Xcalibur 2.1 software (Thermo-Fisher Scientific).

### Protein Identification

Tandem mass spectra from purified CYDV-RPV were converted mascot generic format (MGF) peak list files using Proteome Discovery 1.1. An in-house FASTA protein database was created from all NCBI entries, including all the translations of all the available cereal genomes, common contaminants, and viruses, was downloaded on January 31, 2012. This strategy was used as opposed to restricting the search to green plants to minimize false matches due to the presence of virus gene products or to the presence of other unanticipated sources of proteins (such as from plant-infecting bacteria of fungi) in the samples. All data were searched against this database using Mascot v2.3.02 (Matrix Science, Boston, MA) as follows. Fixed carbamidomethyl and variable methionine oxidation were used as modifications. Precursor ion tolerances were set to 30 parts per million (ppm), and fragment tolerance was 0.8 Dalton (Da). ESI-Trap was selected as the instrument type. The enzyme selected was trypsin with 1 missed tryptic cleavage permitted.

Mascot *.dat files were created in Mascot and loaded into Scaffold (version 3_00_05). Peptide and protein probabilities were calculated using PeptideProphet and ProteinProphet algorithms [90]. We reported protein accession numbers that could be identified on the basis of at least one peptide with a Mascot score exceeding the identity threshold and E-value <0.05. The FDR was less than 1.0%. Spectral counts were normalized to the total and compared between treated and non-treated virions, as well as a healthy control. The healthy control consisted of the fraction in the sucrose gradient corresponding to the virus sedimentation position. Proteins were not reported if they were also detected in the healthy control (Table S1). To minimize redundancy due to effects of homology-based searching, only one protein per protein family were reported. Rubisco was abundant in all virus and healthy plant samples.

### Selected Reaction Monitoring Mass Spectrometry

Parthenogenetically reproducing aphid colonies of *R. padi* were maintained on CYDV-RPV infected or healthy caged oats at 20°C with an 18-h photo-period for 21 days. Aphids were harvested from plants for protein extraction as described [77]. Proteins were extracted from aphids using the TCA-Acetone method as described [77]. The pellets were dried and stored at −80°C until used for mass spectrometry, approximately 2 weeks.

Protein pellets were prepared for mass spectrometry as previously described [80]. Pellets were solubilized by adding a volume of 50 mM ammonium bicarbonate (Sigma; St. Louis, MO)/0.1% RapiGest SF (Waters Corp.; Milford, MA) solution. Samples were left to stir overnight at 4°C and then centrifuged at 16,000×g for 5 min to pellet insoluble debris. Protein concentration of the supernatant was determined using a Quickstart Bradford assay (Biorad) and verified using 1-D SDS PAGE as described [77]. For each sample replicate, 100 µg of protein was diluted in 50 µl 50 mM ammonium bicarbonate/0.1% RapiGest SF (Waters Corp., Milford, MA) and used as the starting material for the digestion procedure. Samples were reduced with DTT at a 5 mM final concentration for 30 min at 50°C and then alkylated with IAA at a 15 mM final concentration for 30 min at room temperature, in the dark. For digestion, a 200 ng/µl trypsin (Promega; Madison, WI) solution was prepared using 0.01% acetic acid. Two µg of trypsin was added to each sample at a trypsin:protein ratio of 1∶50 and incubated at 37°C for 3.5 h, with gentle vortexing every 15 min. To hydrolyze the RapiGest surfactant, samples were acidified with HCl to a pH ≤2, final HCl concentration of 200 mM, incubated at 37°C for 45 min, and centrifuged at 16,000×g for 10 min. The supernatant was transferred to new tubes and frozen at −80°C until mass spectrometry, approximately 48 h. Impurities were removed using mixed mode RP SCX SPE cation exchange cartridges (Waters Oasis 1cc MCX cartridge).

Nano-flow liquid chromatography was performed using an Eksigent 1D nanoLC system (Dublin, CA) with direct column injection. Tips were pulled from silica capillary (75 µm I.D. × 360 µm O.D.) in-house using a commercial CO_2_ laser puller (Sutter Instruments Co., Novato, CA), and then packed to a length of 15 cm with 4 µm C12 reverse phase particles (Phenomenex, Torrance, CA). Two µL of the 1 µg/µL digested aphid protein extracts were injected directly on the column and eluted with a flow-rate of 300 nL/mn. The gradient ramped from 2% B (80∶20 ACN/H_2_0) to 37% B across 50 min, and then increased to 80% B and held constant for 5 min. Initial conditions were restored for the final 15 min of the run. Electrospray ionization (ESI) was initiated by applying 2.2 kV via a liquid junction distally from the ESI tip. The capillary voltage and temperature were 42 V and 275°C, respectively. MS analyses were performed using a TSQ Vantage (ThermoFisher, San Jose, CA) operating in SRM mode. For SRM-mass spectrometry, the doubly charged precursor ions were monitored in Q1 with a resolution of 0.7 full width at half-maximum (FWHM) and three to four singly charged y-ions for each peptide were monitored in Q3 at 0.7 FWHM. Each transition was monitored for 25 ms (dwell time) enabling a maximum duty cycle of 2.5 s.

Targeted protein sequences were imported into Skyline [32] and converted into trypsin fragments. Refinement was performed as described [91]. Briefly, to optimize collection of SRM data, we focused initial analysis on peptides from host proteins that could be detected in the matrix of total protein homogenates extracted from *R. padi* fed on CYDV-RPV infected tissues. From these samples, MS/MS data were collected for Skyline-predicted tryptic peptide ions from host proteins of interest. These data were imported back into Skyline for refinement of the SRM method. During refinement, we selected proteotypic peptides that ionized well (3–4 abundant y-ions for each peptide) and showed reproducible chromatographic retention properties and made a new SRM method. The new, data-driven, refined SRM method was exported to the mass spectrometer. Three biological replicates were analyzed using the refined SRM method, and a Student’s T-test was used to compare total peak areas. A normalization factor of 0.92 was calculated by monitoring for peptides derived from two different proteins that were not differentially expressed between treatments and applied to the peptides derived from healthy samples. Both raw and normalized peak areas for each transition are reported (Table S2).

## Supporting Information

Figure S1 (A) 1-D PAGE of proteins from purified virus showing bands that were excised and digested with trypsin. Lane 1, Broad range molecular weight standards (Biorad) in kDa; Lane 2, transmissible virus; Lane 3; non-transmissible virus. The band containing the RTP is encircled in red. Other bands subjected to LC- MS/MS analysis are indicated with a red *. Multiple proteins were found in each lane. These proteins were abundant contaminants (reported in the text and Table S1) also found in sucrose gradients separating healthy tissue. Tryptic peptides matched to the full-length RTP in (B) non-transmissible virus purified with sodium sulfite and EDTA and (C) transmissible virus are highlighted in red. Virions were purified from the same infected source tissue (oats), the only difference was addition of sodium sulfite and EDTA in homogenization buffer. Peptides were identified from the same region of the protein in both treated and untreated virions indicating no cleavage of the RTP in the non-transmissible virion preparations.
(TIF)

Figure S2 **Clustal W alignment showing CYDV-PRV cysteine residues in the RTP in the context of a multiple alignment of twelve luteovirid species.** C136 and C373 are highly conserved among luteovirids whereas C112 is unique to CYDV-RPV. In CYDV-RPV, all three cysteine residues within the RTP are flanked by at least one basic amino acid, making them especially reactive and likely to be involved in disulfide bonding.
(TIF)

Figure S3 **SRM transitions of the peptides from host plant proteins that were detected in tryptic digests of pooled, whole-body** ***R. padi*** **protein samples (from data shown in** **Table 5** **).** One replicate per peptide is shown. Two peptides from cellulose synthase show no statistically significant fold-change in aphids reared on CYDV-RPV infected or healthy plants (A) SQTGDFDHNR detected aphids fed on CYDV-RPV infected plants or (B) healthy plants, (C) IPMFAYVSR detected in aphids fed on CYDV-RPV infected plants or (D) healthy plants. The peptide FGGDTYCCR from thaumatin-like protein 5 detected in aphids fed on CYDV-RPV infected plants in (E) or healthy plants in (F). The peptide VLYSSCYVR from 33-kD secretory protein detected in aphids fed on CYDV-RPV infected plants (G) or healthy plants (H). The peptide VLYSSCYVR was at the lower limit of detection in the samples derived from aphids reared on healthy plants. The peptide SDSIITAYR from pyruvate dehydrogenase E1 derived from samples of aphids collected from CYDV-RPV infected plants (I) or healthy plants (J). One peptide was detected from pyruvate dehydrogenase E2: GLGMIAEEVK was only detected in samples of aphids reared on CYDV-RPV infected tissues (K), and not in aphids reared on healthy tissue (L). The next step to confirm these differences in aphids fed on healthy or infected plants is validation and absolute quantification of these peptides via the use of stable isotope labeled peptides.
(TIF)

Table S1 **Plant proteins that were identified in the sucrose gradient fractions from healthy oat plants.**
(PDF)

Table S2 **Raw and normalized peak areas, T-test results, and retention time coefficient of variation for plant peptides detected using SRM in aphids fed on CRDV-RPV infected or healthy plants.**
(PDF)
