# Supplementary figures and images for: Discovery and Targeted LC-MS/MS of Purified Polerovirus Reveals Differences in the Virus-Host Interactome Associated with Altered Aphid Transmission
Source: PLoS One. 2012 Oct 30;7(10):e48177. doi: 10.1371/journal.pone.0048177 (PMC3484124; doi:10.1371/journal.pone.0048177)

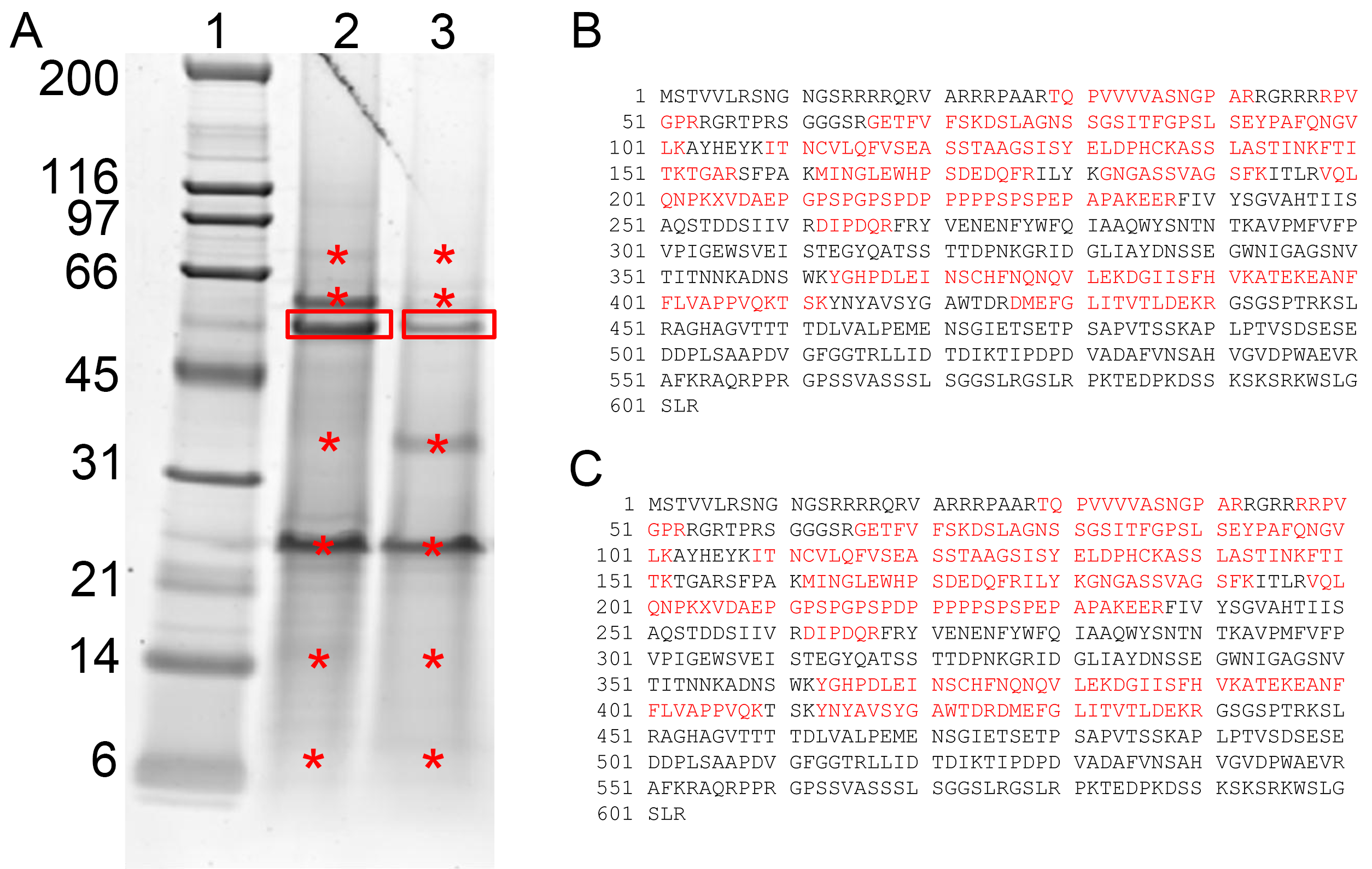

Supplement: Figure S1 — (A) 1-D PAGE of proteins from purified virus showing bands that were excised and digested with trypsin. Lane 1, Broad range molecular weight standards (Biorad) in kDa; Lane 2, transmissible virus; Lane 3; non-transmissible virus. The band containing the RTP is encircled in red. Other bands subjected to LC- MS/MS analysis are indicated with a red *. Multiple proteins were found in each lane. These proteins were abundant contaminants (reported in the text and Table S1) also found in sucrose gradients separating healthy tissue. Tryptic peptides matched to the full-length RTP in (B) non-transmissible virus purified with sodium sulfite and EDTA and (C) transmissible virus are highlighted in red. Virions were purified from the same infected source tissue (oats), the only difference was addition of sodium sulfite and EDTA in homogenization buffer. Peptides were identified from the same region of the protein in both treated and untreated virions indicating no cleavage of the RTP in the non-transmissible virion preparations. (TIF) [file pone.0048177.s001.tif]

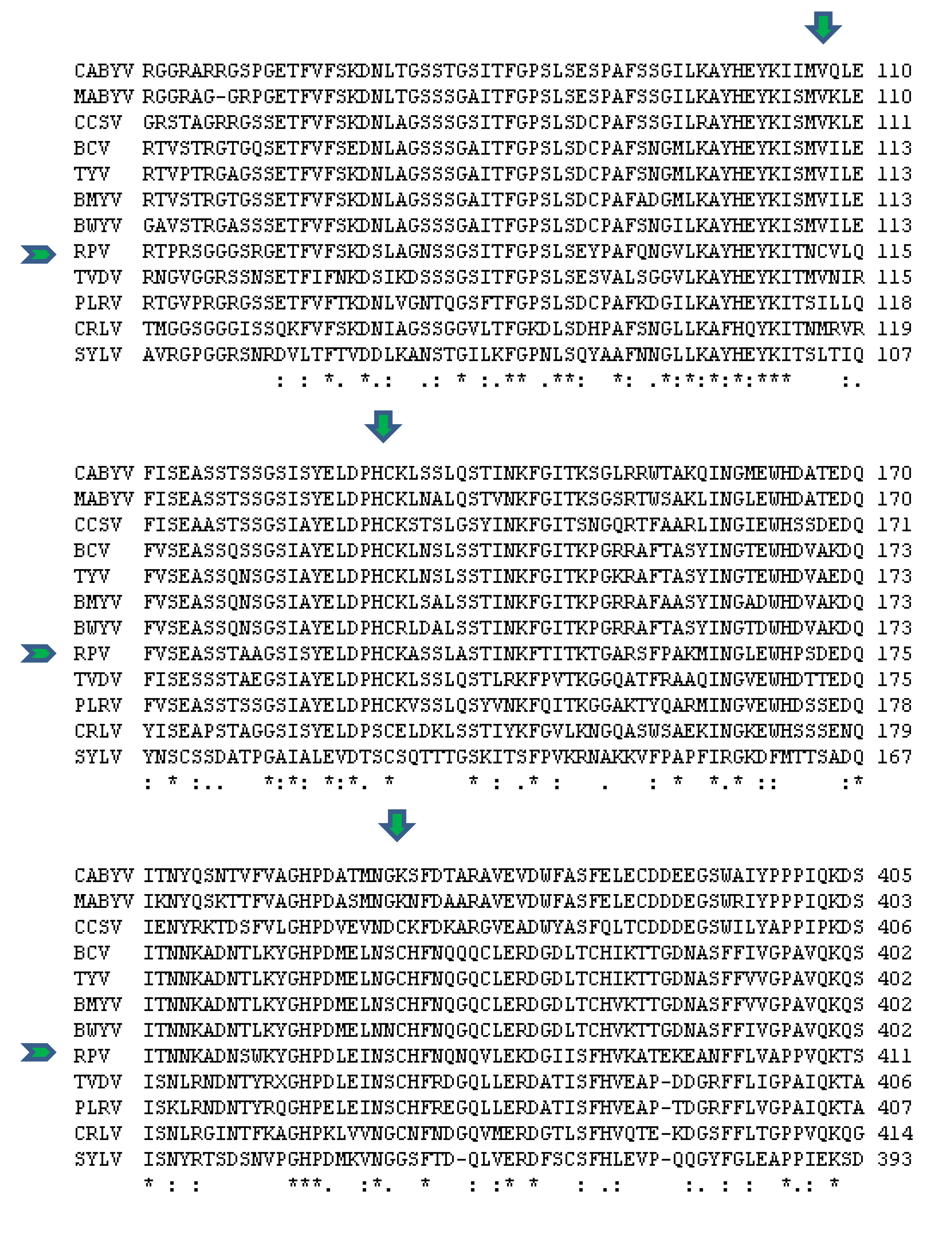

Supplement: Figure S2 — Clustal W alignment showing CYDV-PRV cysteine residues in the RTP in the context of a multiple alignment of twelve luteovirid species. C136 and C373 are highly conserved among luteovirids whereas C112 is unique to CYDV-RPV. In CYDV-RPV, all three cysteine residues within the RTP are flanked by at least one basic amino acid, making them especially reactive and likely to be involved in disulfide bonding. (TIF) [file pone.0048177.s002.tif]

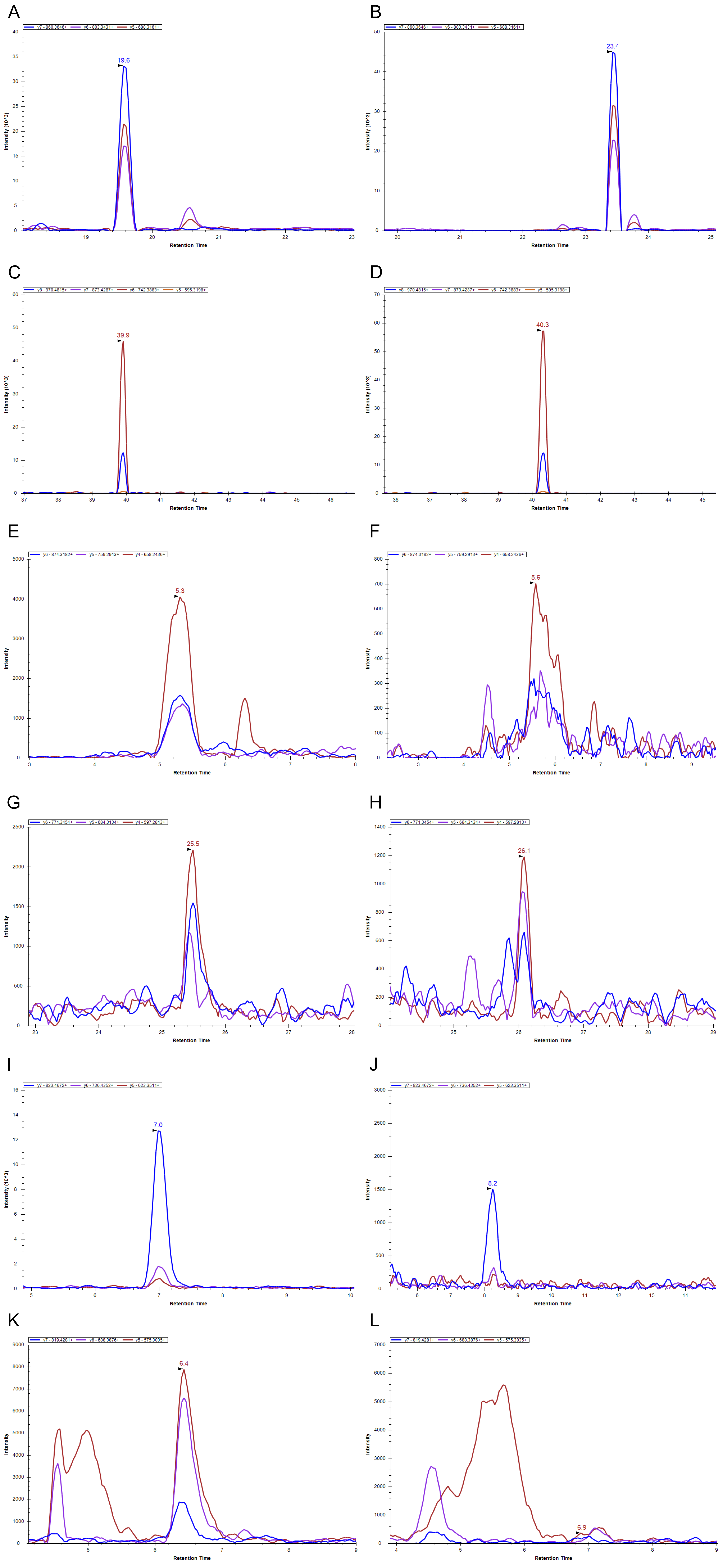

Supplement: Figure S3 — SRM transitions of the peptides from host plant proteins that were detected in tryptic digests of pooled, whole-body R. padi protein samples (from data shown in Table 5 ). One replicate per peptide is shown. Two peptides from cellulose synthase show no statistically significant fold-change in aphids reared on CYDV-RPV infected or healthy plants (A) SQTGDFDHNR detected aphids fed on CYDV-RPV infected plants or (B) healthy plants, (C) IPMFAYVSR detected in aphids fed on CYDV-RPV infected plants or (D) healthy plants. The peptide FGGDTYCCR from thaumatin-like protein 5 detected in aphids fed on CYDV-RPV infected plants in (E) or healthy plants in (F). The peptide VLYSSCYVR from 33-kD secretory protein detected in aphids fed on CYDV-RPV infected plants (G) or healthy plants (H). The peptide VLYSSCYVR was at the lower limit of detection in the samples derived from aphids reared on healthy plants. The peptide SDSIITAYR from pyruvate dehydrogenase E1 derived from samples of aphids collected from CYDV-RPV infected plants (I) or healthy plants (J). One peptide was detected from pyruvate dehydrogenase E2: GLGMIAEEVK was only detected in samples of aphids reared on CYDV-RPV infected tissues (K), and not in aphids reared on healthy tissue (L). The next step to confirm these differences in aphids fed on healthy or infected plants is validation and absolute quantification of these peptides via the use of stable isotope labeled peptides. (TIF) [file pone.0048177.s003.tif]
