# Supplementary material for: Discovery and Targeted LC-MS/MS of Purified Polerovirus Reveals Differences in the Virus-Host Interactome Associated with Altered Aphid Transmission
Source: PLoS One. 2012 Oct 30;7(10):e48177. doi: 10.1371/journal.pone.0048177 (PMC3484124; doi:10.1371/journal.pone.0048177)
Supplement: Table S1 — Plant proteins that were identified in the sucrose gradient fractions from healthy oat plants. (PDF) [file pone.0048177.s004.pdf]

Table S1. Plant proteins found in sucrose gradient fractions from healthy plants, Protein ID % probability >99%

NCBI Protein Name	NCBI Accession	BLAST Annotation
HIS4; DNA binding [Arabidopsis thaliana]	gi|15226944	
beta-D-glucosidase [Avena sativa]	gi|505279	
predicted protein [Hordeum vulgare subsp. vulgare]	gi|326500636	ribosomal protein S4
RecName: Full=Ribulose bisphosphate carboxylase large chain; Short=RuBisCO large subunit; Flags: Precursor	gi|1346964	
predicted protein [Hordeum vulgare subsp. vulgare]	gi|326497321	putative pyrophosphate-dependent phosphofructo-1-kinase
predicted protein [Hordeum vulgare subsp. vulgare]	gi|326532144	putative pyrophosphate-dependent phosphofructo-1-kinase
predicted protein [Hordeum vulgare subsp. vulgare]	gi|326499005	histone 2A
PREDICTED: glyceraldehyde-3-phosphate dehydrogenase B, chloroplastic-like [Brachypodium distachyon]	gi|357114230	
PREDICTED: 6-phosphofructokinase 3-like [Brachypodium distachyon]	gi|357132904	
beta-D-glucosidase beta subunit precursor [Avena sativa]	gi|4106413	
Chain E, Localization Of The Large Subunit Ribosomal Proteins Into A 5.5 A Cryo-Em Map Of Triticum Aestivum Translating 80s Ribosome	gi|313103578	
photosystem I subunit VII [Zygnema circumcarinatum]	gi|108796706	
predicted protein [Hordeum vulgare subsp. vulgare]	gi|326496288	40S ribosomal protein S11
PREDICTED: 60S ribosomal protein L6-like [Brachypodium distachyon]	gi|357140576	
Os12g0567700 [Oryza sativa Japonica Group]	gi|115489150	60S ribosomal protein L2
Os01g0679700 [Oryza sativa Japonica Group]	gi|115439177	60S ribosomal protein L37a
putative ribosomal protein L26 [Oryza sativa Japonica Group]	gi|12328551	
PREDICTED: 40S ribosomal protein S18-like [Brachypodium distachyon]	gi|357111429	
predicted protein [Hordeum vulgare subsp. vulgare]	gi|326488173	60S ribosomal protein L4
Chain i, Localization Of The Large Subunit Ribosomal Proteins Into A 5.5 A Cryo-Em Map Of Triticum Aestivum Translating 80s Ribosome	gi|313103611	
Os02g0287000 [Oryza sativa Japonica Group]	gi|115445597	40S ribosomal protein S3a
ubiquitin [Lolium temulentum]	gi|168472729	
Os09g0502000 [Oryza sativa Japonica Group]	gi|115479981	60S ribosomal protein L32-1-like
hypothetical protein LOC100193262 [Zea mays]	gi|212722360	40S ribosomal protein S26
Chain k, Localization Of The Large Subunit Ribosomal Proteins Into A 5.5 A Cryo-Em Map Of Triticum Aestivum Translating 80s Ribosome	gi|313103613	
predicted protein [Hordeum vulgare subsp. vulgare]	gi|326488131	40S ribosomal protein S8
predicted protein [Hordeum vulgare subsp. vulgare]	gi|326500108	50S ribosomal protein L24, chloroplastic-like
beta-D-glucan exohydrolase, isoenzyme ExoII [Hordeum vulgare subsp. vulgare]	gi|1203832	
Os01g0348700 [Oryza sativa Japonica Group]	gi|115436432	60S ribosomal protein L23a
PREDICTED: serine hydroxymethyltransferase, mitochondrial-like [Brachypodium distachyon]	gi|357116394	
Os07g0622100 [Oryza sativa Japonica Group]	gi|115473429	40S ribosomal protein S6
fructose-bisphosphate aldolase, putative [Ricinus communis]	gi|255581400	
predicted protein [Hordeum vulgare subsp. vulgare]	gi|326489899	50S ribosomal protein L15, chloroplastic-like
Os01g0896800 [Oryza sativa Japonica Group]	gi|115441607	60S ribosomal protein L5-1
RecName: Full=60S ribosomal protein L10	gi|18203270	
unknown [Picea sitchensis]	gi|116781756	60S ribosomal protein L13a-4-like
PREDICTED: 40S ribosomal protein S14-like [Brachypodium distachyon]	gi|357123442	
PREDICTED: 40S ribosomal protein SA-like [Brachypodium distachyon]	gi|357121904	
Os02g0137200 [Oryza sativa Japonica Group]	gi|115444097	50S ribosomal protein L3, chloroplastic-like
PREDICTED: ruBisCO large subunit-binding protein subunit alpha, chloroplastic-like [Brachypodium distachyon]	gi|357167236	
ribosomal protein L2 [Oryza sativa Indica Group]	gi|109156625	
Ribulose-1,5-bisphosphate carboxylase/oxygenase [Mitrasacme pilosa]	gi|6688796	
cysteine protease 1 [Zea mays]	gi|226496089	
ribulose bisphosphate carboxylase oxygenase [Stenanthemum pomaderroides]	gi|9968778	
fructose-1,6-bisphosphatase, cytosolic,putative,expressed [Triticum aestivum]	gi|300681469	
PREDICTED: uncharacterized protein LOC100835762 [Brachypodium distachyon]	gi|357112407	Lectin binding protein
