# Supplementary material for: Discovery and Targeted LC-MS/MS of Purified Polerovirus Reveals Differences in the Virus-Host Interactome Associated with Altered Aphid Transmission
Source: PLoS One. 2012 Oct 30;7(10):e48177. doi: 10.1371/journal.pone.0048177 (PMC3484124; doi:10.1371/journal.pone.0048177)
Supplement: Table S2 — Raw and normalized peak areas, T-test results, and retention time coefficient of variation for plant peptides detected using SRM in aphids fed on CRDV-RPV infected or healthy plants. (PDF) [file pone.0048177.s005.pdf]

Table S2. Peak areas, T-test results, and retention time coefficient of variation for plant peptides detected in aphids fed on CRDV-RPV infected or healthy plants (reported in Table 5)

ReplicateName[a]	Sequence	RT[b]	Average RT	Precursor Mz	Total Area[c]	Normalized Area	Fold-change[d]	Log2 Fold Change	T-test	RT CV (%)
RPV_neg_RPVhost	SQTGDFDHNR	21.89	22.05	588.755058	1015623	934373.16	0.788945426	-0.342002587	0.466257	9.954705
RPV_neg_RPVhost2	SQTGDFDHNR	23.47			833912	767199.04				
RPV_neg_RPVhost3	SQTGDFDHNR	25.65			430855	396386.6				
RPV_plus_RPVhost	SQTGDFDHNR	21.09			466487					
RPV_plus_RPVhost2	SQTGDFDHNR	19.57			708443					
RPV_plus_RPVhost3	SQTGDFDHNR	20.63			480245					
RPV_neg_RPVhost	IPMFAYVSR	40.34	40.44	542.286419	836229	769330.68	1.007770703	0.011167421	0.967548	1.737617
RPV_neg_RPVhost2	IPMFAYVSR	41.03			427102	392933.84				
RPV_neg_RPVhost3	IPMFAYVSR	41.53			754913	694519.96				
RPV_plus_RPVhost	IPMFAYVSR	39.91			589209					
RPV_plus_RPVhost2	IPMFAYVSR	39.71			575426					
RPV_plus_RPVhost3	IPMFAYVSR	40.11			706578					
RPV_neg_RPVhost	FGGDTYCCR	5.54	5.46	568.218408	43128	39677.76	4.07839288	2.02800076	0.013361	6.139949
RPV_neg_RPVhost2	FGGDTYCCR	5.67			44452	40895.84				
RPV_neg_RPVhost3	FGGDTYCCR	5.97			32503	29902.76				
RPV_plus_RPVhost	FGGDTYCCR	5.11			137250					
RPV_plus_RPVhost2	FGGDTYCCR	5.11			143753					
RPV_plus_RPVhost3	FGGDTYCCR	5.38			169563					
RPV_neg_RPVhost	VLYSSCYVR	26.09	25.95	573.784241	26247	24147.24	3.122682746	1.642786004	NC	1.77628
RPV_neg_RPVhost2	VLYSSCYVR	26.68			25214	23196.88				
RPV_neg_RPVhost3	VLYSSCYVR	26.12			ND	ND				
RPV_plus_RPVhost	VLYSSCYVR	25.46			51534					
RPV_plus_RPVhost2	VLYSSCYVR	25.46			74584					
RPV_plus_RPVhost3	VLYSSCYVR	25.92			95643					
RPV_neg_RPVhost	SDSIITAYR	7.72	7.48	513.266738	42205	38828.6	6.24597266	2.642926254	0.000363	6.278016
RPV_neg_RPVhost2	SDSIITAYR	7.68			33478	30799.76				
RPV_neg_RPVhost3	SDSIITAYR	8.21			45702	42045.84				
RPV_plus_RPVhost	SDSIITAYR	7.06			231763					
RPV_plus_RPVhost2	SDSIITAYR	7.02			233367					
RPV_plus_RPVhost3	SDSIITAYR	7.19			232384					
RPV_neg_RPVhost	GLGMIAEEVK	6.96	6.63	523.781167	ND	ND	NC	NC	NC	2.264119
RPV_neg_RPVhost2	GLGMIAEEVK	6.56			ND	ND				
RPV_neg_RPVhost3	GLGMIAEEVK	6.83			ND	ND				
RPV_plus_RPVhost	GLGMIAEEVK	6.4			269335					
RPV_plus_RPVhost2	GLGMIAEEVK	6.4			288705					
RPV_plus_RPVhost3	GLGMIAEEVK	6.66			333112					

a: RPV_Plus samples are proteins collected from aphids reared on RPV infected plants, RPV_neg samples are proteins from aphids reared on healthy plants
b: measured retention time (RT)
c: peak areas as reported or not detected (ND)
d: fold-change calculated as peak areas of RPV/healthy samples or not calculated (NC)
